# Evaluation of Bone Wax Coated Bipolar Coagulation Forceps: Performance and Safety Assessment

**DOI:** 10.3389/fsurg.2021.816295

**Published:** 2022-01-20

**Authors:** Jichun Shi, Wei Wei, Zhen Wang, Haobin Ren, Chenguang Jia, Lixin Dong, Zhengwei Li, Jianjian Zhang, Yu Feng, Kaixin Huang, Xiang Li, Jincao Chen

**Affiliations:** ^1^Department of Neurosurgery, Zhongnan Hospital of Wuhan University, Wuhan, China; ^2^Brain Research Center, Zhongnan Hospital of Wuhan University, Wuhan, China; ^3^Cognitive Neuroepigenetics Laboratory, Queensland Brain Institute, The University of Queensland, Brisbane, QLD, Australia; ^4^Frontier Science Center for Immunology and Metabolism, School of Medicine, Medical Research Institute, Wuhan University, Wuhan, China

**Keywords:** bipolar forceps, bone wax, coagulation, electrocoagulation, hemostasis, hemostatic

## Abstract

**Background:**

Improving the performance of bipolar coagulation forceps is crucial for safer and more accurate neurosurgery. In our department, we found that bone wax (BW) melted by thermal effect of bipolar electrocoagulation can achieve more efficient hemostasis and reduce the amount of BW in neurosurgical procedures associated with bleeding from emissary and diploic veins. Nevertheless, relevant studies are still lacking to verify our finding.

**Objective:**

The study objectives were to evaluate the performance and safety in electrocoagulation: (1) compare the performance of BW coated bipolar coagulation forceps and the conventional anti-stick forceps *in vivo*, and (2) assess the safety of electrocoagulation with BW coated bipolar coagulation forceps in rat primary motor cortex.

**Methods:**

Tissue adhesion was evaluated by comparing the wetting tension and the amount of protein adhered to the forceps tips after electrocoagulation. Thermal damage was assessed by analyzing the thermography and H&E staining of coagulated rat brain tissues. The hemostatic efficiency was reflected by the number of electrocoagulation until complete hemostasis and the condition of damaged common carotid arteries. The safety of BW coated forceps in electrocoagulation was assessed by evaluating the inflammation of coagulated rat primary motor cortex and the motor functions at the 7th day postoperatively.

**Results:**

Bone wax coated forceps had a significantly higher contact angle and adhered less coagulum. Thermography was acquired at 3 s, 6 W units in rat primary motor cortex *in vivo*. The highest temperature recorded during BW coated tips application was significantly lower than the uncoated. In addition, there was a relatively smaller tissue injury area produced by the BW coated forceps. Additionally, BW coated forceps improved the hemostatic efficiency and caused fewer injuries on the damaged arteries (3 s, 10 W units). More importantly, electrocoagulation with BW coated forceps led to no significant motor function impairments and less glial and microglia responses.

**Conclusion:**

This study reveals that BW coated bipolar coagulation forceps can provide a convenient, cost-efficient, safer, and more efficient way for hemostasis. More research is needed to evaluate the electrocoagulation with BW in the long term and verify our finding in human beings.

## Introduction

Intraoperative hemorrhage is likely to be the most severe situation arising in neurosurgical procedures, associated with severe morbidity and mortality (1). Adequate hemostasis is a prerequisite to keep operative fields clean and smooth subsequent surgical manipulations, which is imperative for patients undergoing neurosurgical intervention. Generally, the hemostatic methods can be divided into 3 categories: mechanical, chemical, and thermal (2). Each of these methods has its advantages and disadvantages due to their different indications. The combination of different mechanisms may provide synergistic effects in hemostasis.

Bipolar electrocoagulation, first introduced in 1940 by James Greenwood (3), is a basic hemostatic technique of modern neurosurgery. The electrocoagulation is achieved by delivering high-frequency alternating current into living tissue. As tissues resist the current, electrical energy can be converted into heat. The thermal effect on tissues between bipolar electrodes evaporates water and cauterizes the tissue to achieve hemostasis (4). In most cases, electrocoagulation has proven to be a reliable hemostatic method. Whereas, the application of bipolar coagulation is still difficult to control bleeding when dealing with difficult conditions, such as ruptured vessels with structural defects, hemorrhage of tissues with high blood flow, and vital centers. Furthermore, tissue sticking, charring, and thermal injury are still the major concerns in bipolar electrocoagulation, which may result in unintended bleeding or neuronal damage. All drawbacks above will make the operative process less accurate and more time-consuming (5). To overcome these drawbacks, approaches, such as irrigation and new tip technologies have been developed to improve the electrocoagulation performance (5–7). However, these approaches solved only part of the problem and themselves created new problems. They may impair hemostatic effect, cause severe cell cytotoxicity, or have high application cost. Hence, for dealing with difficult hemostasis situations, cost-effective, better performing, and safe improvements for electrocoagulation forceps should be an important matter for earnest development.

Incidentally, in neurosurgical procedures associated with bleeding from emissary and diploic veins, we found that bone wax (BW) melted by the electrocoagulation thermal effect can achieve more efficient hemostasis and reduce the used amount. Additionally, based on the hypothesis that a combination of different mechanisms could improve hemostatic efficiency and ameliorate drawbacks caused by bipolar electrocoagulation, and then BW came to our minds. Currently, there are still no relevant studies to verify our speculation. BW, a sterile, non-absorbable mixture of beeswax (70%) and Vaseline (30%), is the most used bony hemostatic agent. The main hemostatic action is relied on its physical property, which act as a tamponade by stopping the blood flow from damaged vessels in the trabecular bone and thereby facilitating clot formation. Nevertheless, BW may cause slight complications, such as inflammation in bone because of its neither absorbed nor metabolized property (8–10). In addition, the current speculation about forming foreign body granuloma in brain comes from several clinical case reports about “BW migration” with the use of BW in a large quantity (10, 11). Thus, it is necessary to evaluate the safety of a small amount of BW in brain. Taking advantage of thermal effect, BW can be melted to seal ruptured blood vessels. Furthermore, based on the mechanical and thermal dual hemostatic mechanisms, we supposed that bipolar forceps tips coated with a small amount of BW might provide a better hemostatic method.

In this study, owing to the quest for more efficient and safer bipolar electrocoagulation, we assessed performance and safety of the novel hemostatic method, bipolar forceps coated with small amount of BW, during the experimental electrocoagulation in rats.

## Materials and Methods

### Bipolar Coagulation

**Figure 1:**
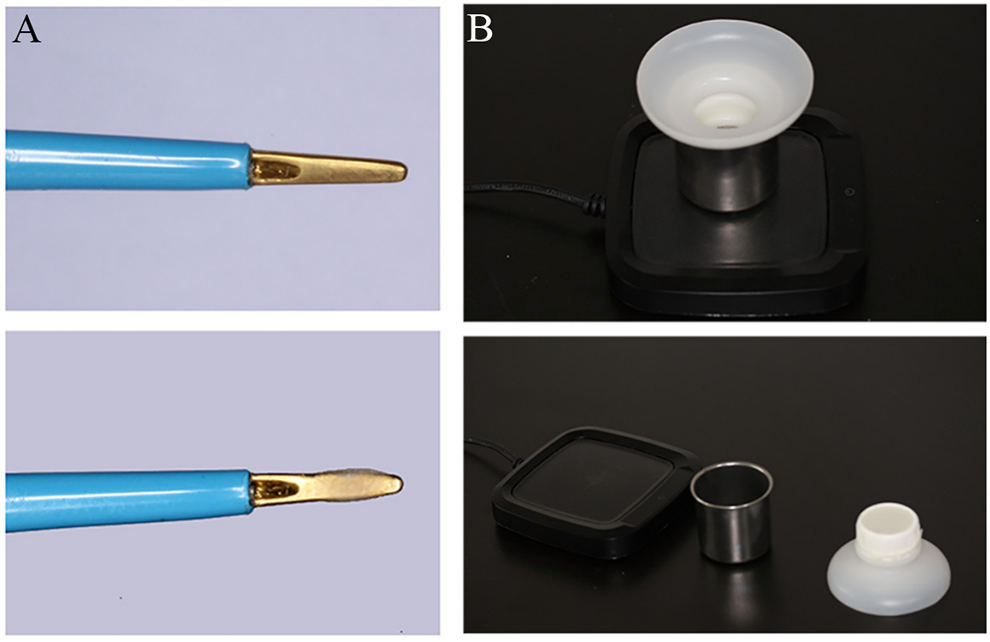
Photograph depicting bone wax (BW) coated, uncoated bipolar forceps **(A)** and BW applied device **(B)**.

Bone wax (Knochenwachs; Melsungen, Germany) coated and uncoated disposable bipolar forceps (GOLDBOV JBW/D-D, Wuhan, China) were assessed in our study (Figure 1A). To facilitate the application of small amount of BW in electrocoagulation, we developed a simple device (Figure 1B). The device consisted of three parts, such as constant temperature heating device (~60°C to melt the BW), steel cup (to load BW), and filtering device with elastic mesh (to coat BW evenly and remove the excess). It is very convenient to use, just dip the forceps tips through the elastic mesh into the melted BW and take it out. In the following study, the electrocoagulation was carried out with a Malis bipolar coagulator (Covidien Force FX-8C; MA, USA).

### Assessment of Adherence

Wetting tension, a measurable property that estimates the surface energy of a film, is the maximum liquid surface tension that will spread on the film surface rather than bead up (12). It is specific for any given system and reflected by measuring the contact angle, which is a measurement of the behavior of pure water in contact with the film surface and can reflect the surface hydrophobicity. The larger contact angle indicates stronger hydrophobicity, related to the lower surface adhesion. To determine the wetting tension, the contact angle of a pure water drop (2 μl) on the surface of the BW coated and uncoated tips was measured at 25°C in air saturated with moisture by Drop Shape Analyzer (DSA100S, KRÜSS; Hamburg, Germany). Measurement was repeated six times, respectively.

As reported previously (12, 13), whole blood from experimental rats was used to evaluate adherence in our study. In the study, 100 μl of the whole blood preincubated at 37°C was placed in a 1.5 ml Eppendorf tube, which was just overwhelming the forceps tips. The electrocoagulation was performed at the constant time and electric power (5 s, 6 W units). After coagulation, the adhered coagulum was completely removed by an ultrasonic processor (TINME CDS-300; Munich, Germany) in the solution of 1 ml 1% Triton-X 100, and the protein content of the solution was assayed by a bicinchoninic acid protein assay reagent (Thermo Fisher 23227, MA, USA). The coagulation at each condition was repeated six times and the amount of coagulum was expressed as the average protein amount in these six procedures.

### Animals

The protocols for animal experiments were reviewed and approved by the Institutional Animal Care and Use Committee. In this study, 22 Sprague–Dawley male rats (6–8 weeks, weight 200–250 g, China Three Gorges University, China) were maintained in accordance with the guidelines for the care and use of laboratory animals. The animals were fed *ad libitum* and housed in an environment controlled for temperature, humidity, and light. There was no selective bias between the rats operated with the BW coated and uncoated bipolar forceps.

Anesthesia was performed by injecting 1.5% pentobarbital sodium (30 mg/kg) intraperitoneally and maintained with spontaneous ventilation. Surgeries were performed under aseptic conditions using an operating microscope (MOELLER CORELLA XY, Wedel, Germany). All rat surgeries in the whole study were performed by the same skilled researcher.

### Thermography and Thermal Damage Assessment

According to Paxinos and Watson (14) rat brain atlas (14), the bilateral surfaces of brain primary motor cortex (M1) was exposed through generous craniotomies. Briefly, the scalp was incised along the midline, and skull was exposed. We made an approximate 5 × 5mm triangular bone window, and the distance from the inner inferior point to the bregma was 1 mm in length and width. After picking up the dura mater, the bilateral M1 were exposed. Coagulated lesions on the surface of M1 (BW uncoated forceps for the left; BW coated forceps for the right) were created using the same activation time and power setting (3 s, 6 W units) for the *in vivo* experiments (*n* = 6). Bipolar coagulation with each instrument was applied under microscopic visualization with the forceps tips held 1 mm apart and approximately 2 mm deep into the brain. An infrared camera (FLIR C2 thermal camera, OR, USA) was used to collect temperature data. The obtaining thermographs were analyzed by the FLIR Tools computer program.

Subsequently, the brains were collected from rats (*n* = 3), fixed in paraformaldehyde, paraffin-embedded, and coronally sectioned (4 μm) through the area of the brain lesions for H&E staining. Thermal damage was assessed by measuring the injured cross-sectional area and quantified by Image J analysis software (NIH Image, Bethesda, MD, USA).

### Evaluation of the Hemostatic Efficiency

The bilateral common carotid arteries (CCAs) were exposed by placing a piece of surgical glove beneath them. The proximal and distal sides were temporarily blocked with vascular clips to reduce the influence of blood flow. Approximately 1 mm longitudinal incision was made by micro scissors. BW coated and uncoated bipolar forceps were used for electrocoagulation (3 s, 10 W units). Then, the proximal vascular clip was released to determine whether the complete hemostasis was achieved. Otherwise, electrocoagulation was repeated. The number of electrocoagulation was recorded and compared. The coagulated arteries were resected axially, followed by histological studies to compare the damage difference. Histological studies included H&E and oil red O staining for frozen sections (6–8 μm). Experiments were repeated four times (*n* = 4).

### Evaluation of the Safety of BW Coagulation in the Brain

Anesthesia and M1 exposure were the same as above. Differently, to facilitate the tests of motor function impairments, only left M1 was exposed. BW coated and uncoated bipolar forceps were used for electrocoagulation (3 s, 6 W units). No electrocoagulation was on sham group (*n* = 4 for each group, total 12 rats). Body weight change was recorded. At the 7th day postoperatively, a series of behavioral tests, such as open field grid test, grip strength test, and ladder test (tail load for 50 g, 2 cm for ladder interval, and 1 m for ladder length), were conducted to quantify potential motor impairments. The main testing procedures were according to the previous report (15). In open field grid test, total traveled distance and average speed was measured. Grip strength and number of right paw slips were recorded through grip strength and ladder tests, respectively. After evaluation of the motor functions, rats were sacrificed for histology study to determine the inflammation, which was based on a previously described method (16). Briefly, antibody GFAP (Abcam, ab7260, Cambridge, UK) (represented for activated astrocytes) and IbaI (Abcam, ab178847) (represented for activated microglia) were used to visualize the activated microglia and astrocytes in coagulated area for immunofluorescence staining.

### Statistics

Data were expressed as mean ± SEM. The 2-tailed unpaired Student's *t*-test and analysis of variance (ANOVA) with Tukey's multiple comparisons were applied using GraphPad Prism v9.01 software. Analysis of two way-ANOVA was used to analyze the body weight change difference in different groups. Values of ^*^p < 0.05 were considered significant.

## Results

### Assessment of Adherence

**Figure 2:**
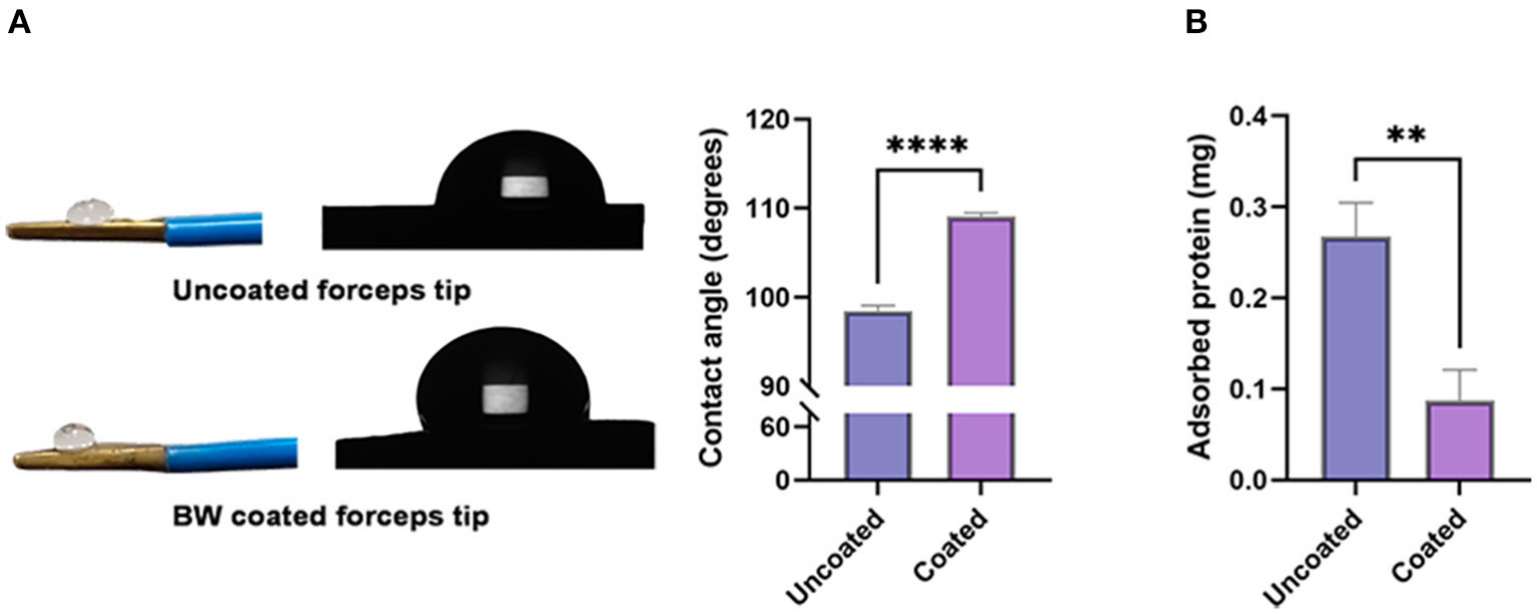
Contact angle and electrocoagulation adherence of BW coated and uncoated tip. [**(A)**-left] Photograph and schematic diagram of a 2 μl water drop on BW coated and uncoated tips. [**(A)**-right] Bar graph revealing the value of contact angle (degrees) (*n* = 6). **(B)** Absorbed protein of BW coated and uncoated tips (*n* = 6). A 2-tailed unpaired Student's *t*-test, **p < 0.01, ****p < 0.0001.

Figure 2A -left shows 2 μl water drop on the forceps surface. The average contact angles differed: 98.47 ± 0.6161 degrees for the uncoated forceps tip, and 109.1 ± 0.4092 degrees for the BW coated forceps tip (^****^ *p* < 0.0001) (Figure 2A -right). Adherence of coagulum to the tips during electrocoagulation, expressed by its adhered protein content, was compared. The amount of adhered protein was significantly less on BW coating forceps tips (uncoated: 0.2672 ± 0.03755 mg vs. coated: 0.08710 ± 0.03389 mg, ^**^ *p* = 0.0052) (Figure 2B).

### Thermography and Thermal Damage Assessment

**Figure 3:**
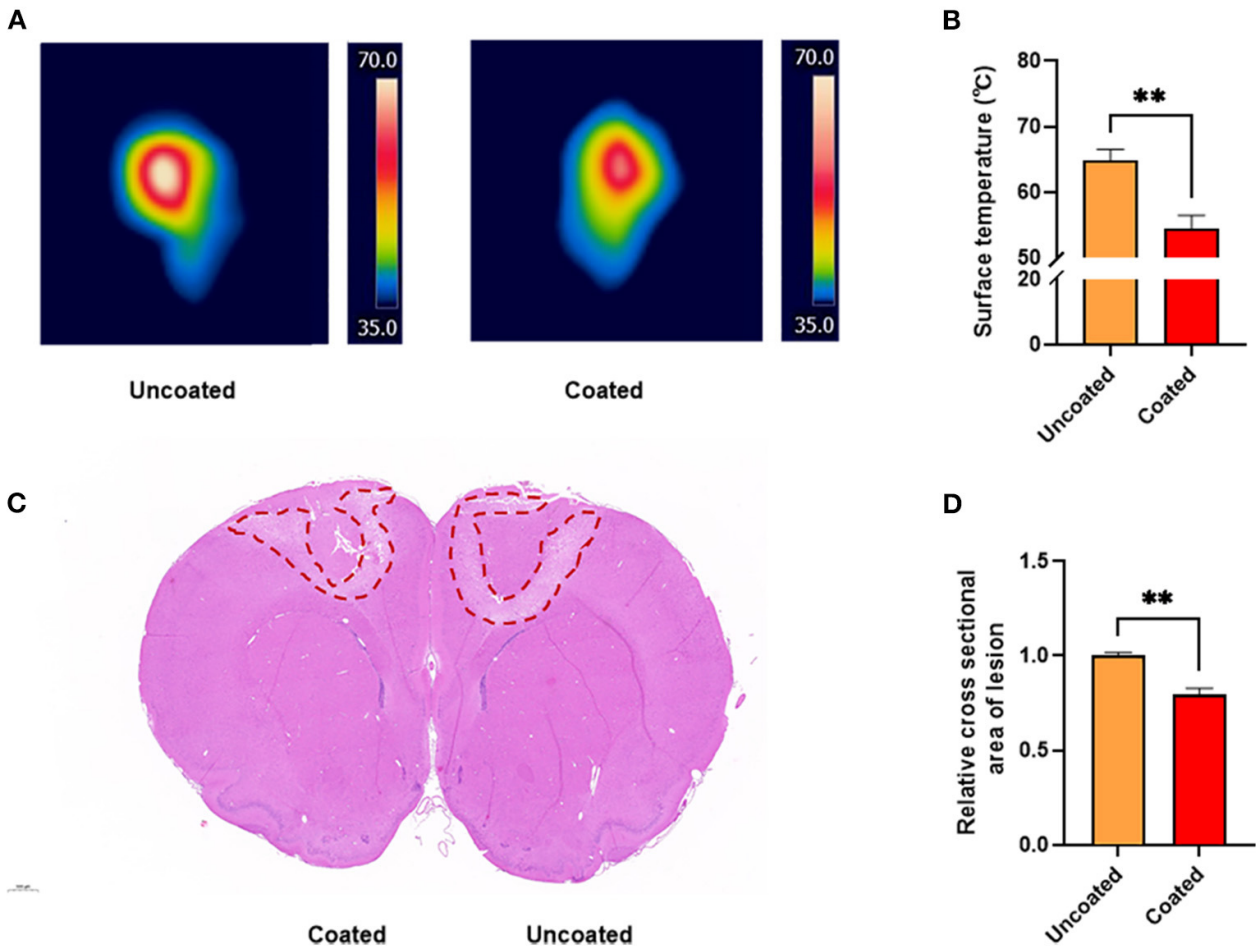
Thermography and thermal damage assessment of the rat primary motor cortex. **(A)** Thermal images acquired at 3 s, 10 W unit *in vivo* rat primary motor cortex studies. **(B)** Bar graph revealing the highest temperature recorded during electrocoagulation (*n* = 6). **(C)** H&E staining of the cross section of coagulated brain. **(D)** Bar graph of the relative cross section damaged areas (*n* = 3). A 2-tailed unpaired Student's *t*-test, **p < 0.01.

Thermographs were obtained (Figure 3A), which illustrated that the tissue surface reached higher temperatures with the conventional anti-stick forceps (Figure 3B). The highest temperature while applying uncoated tips was 64.9 ± 1.67°C, which was significantly higher than the temperature recorded using BW coated tips (54.5 ± 2.02°C, ^**^ *p* = 0.0027).

In addition, based on the analysis of the coagulated damage area marked by H&E staining (Figure 3C), we found that BW coated tips significantly alleviated collateral damage to the brain by approximately 20% compared with the uncoated (^**^ *p* = 0.0046) (Figure 3D). The result suggested BW coated bipolar forceps tips caused less thermal injury.

### Evaluation of the Hemostatic Efficiency

**Figure 4:**
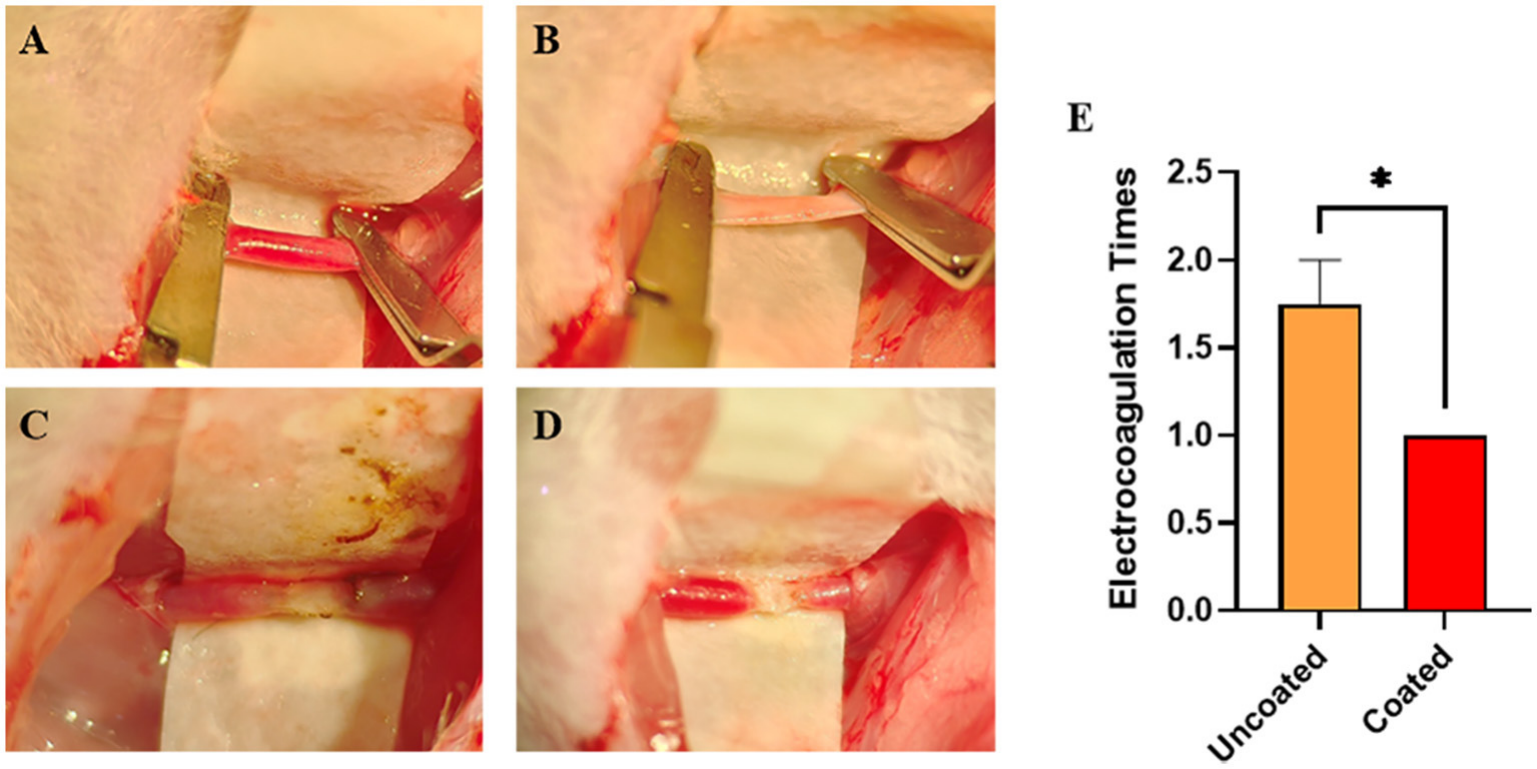
Electrocoagulation condition of BW coated and uncoated bipolar forceps till the complete hemostasis of damaged common carotid arteries (CCAs). **(A)** Intact CCAs. **(B)** Damaged CCA with an approximately 1 mm longitudinal incision. **(C)** Completely hemostasis with uncoated bipolar forceps. **(D)** Complete hemostasis with BW coated bipolar forceps. **(E)** Graph of the number of electrocoagulation with BW coated and uncoated bipolar forceps (*n* = 4). A 2-tailed unpaired Student's *t*-test, *p < 0.05.

Intending to control bleeding of the damaged CCAs (Figures 4A,B), electrocoagulation was applied till the complete hemostasis was achieved. Comparing with the uncoated, BW coated bipolar forceps caused less carbonization and injury (Figures 4C,D). The number of electrocoagulation with BW coated forceps was significantly less (^*^ *p* = 0.0240) (Figure 4E; Table 1), revealing that BW coated forceps were more effective in hemostasis than the conventional anti-stick forceps.

**Table 1:** Electrocoagulation condition of bone wax (BW) coated and uncoated forceps till the complete hemostasis of damaged common carotid arteries (CCAs).

**Rats**	**Number of electrocoagulation**	**Conditions**
**Uncoated bipolar forceps**		
1	2	1-Staxis; 2-Complete hemostasis
2	1	Complete hemostasis
3	2	1-Staxis; 2-Complete hemostasis
4	2	1-Staxis; 2-Complete hemostasis
**Coated bipolar forceps**		
1	1	Complete hemostasis
2	1	Complete hemostasis
3	1	Complete hemostasis
4	1	Complete hemostasis

**Figure 5:**
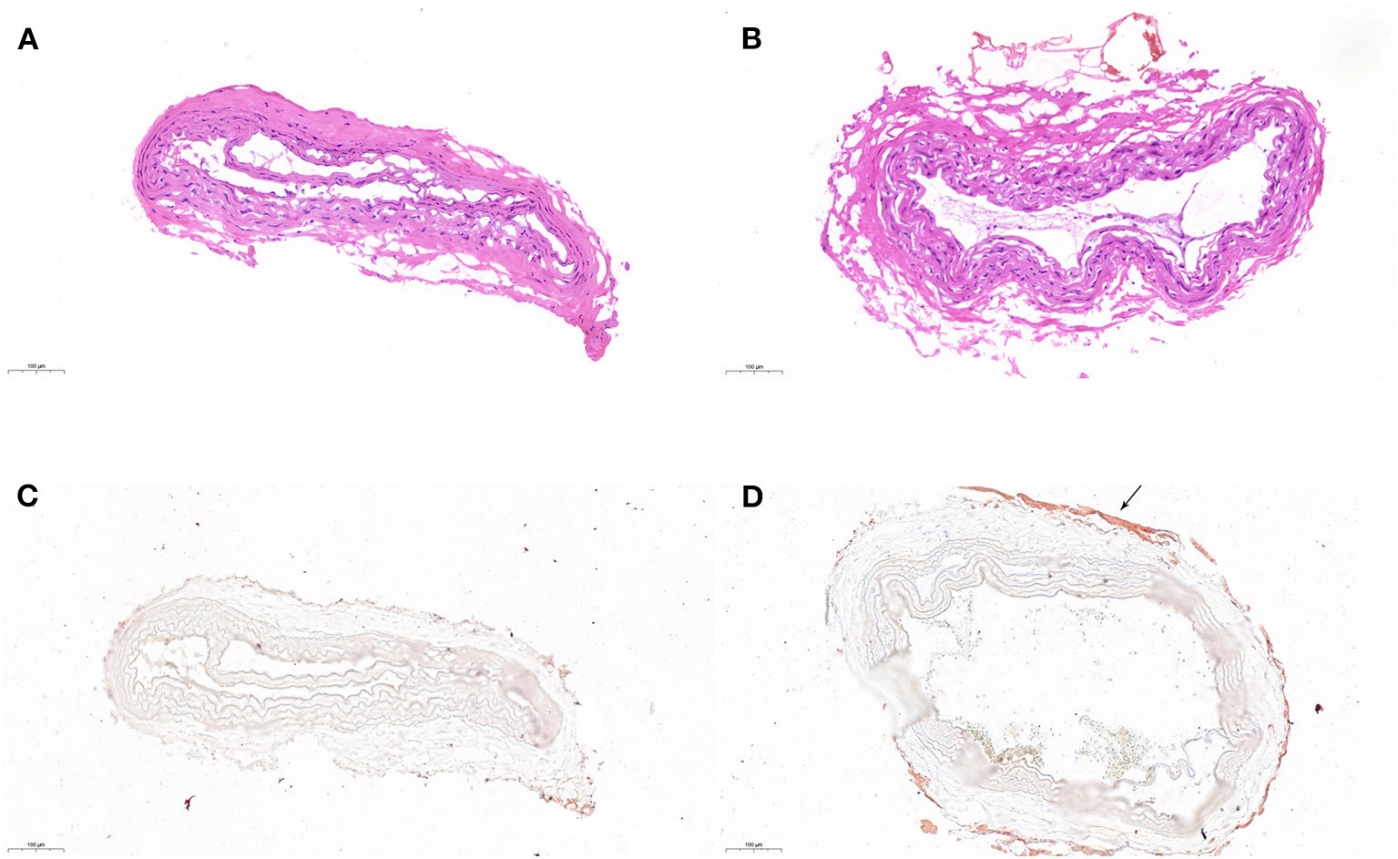
Histological examination of coagulated CCA. H&E staining of coagulated CCA with uncoated **(A)** and BW coated **(B)** bipolar forceps. Oil red O staining of coagulated CCA with uncoated **(C)** and BW coated **(D)** bipolar forceps. The waxy barrier was pointed with the arrow.

With the further histological examination, electrocoagulation with the uncoated caused severe damage to the vascular structure and disturbed the intimal layer of CCA (Figure 5A). The structure was almost intact when using BW coated forceps (Figure 5B). Moreover, according to oil red O staining, electrocoagulation with BW coated forceps formed a waxy barrier to promote hemostasis, which did not appear in the uncoated one (Figures 5C,D).

### Safety Evaluation of Electrocoagulation in M1 Area of Rats

**Figure 6:**
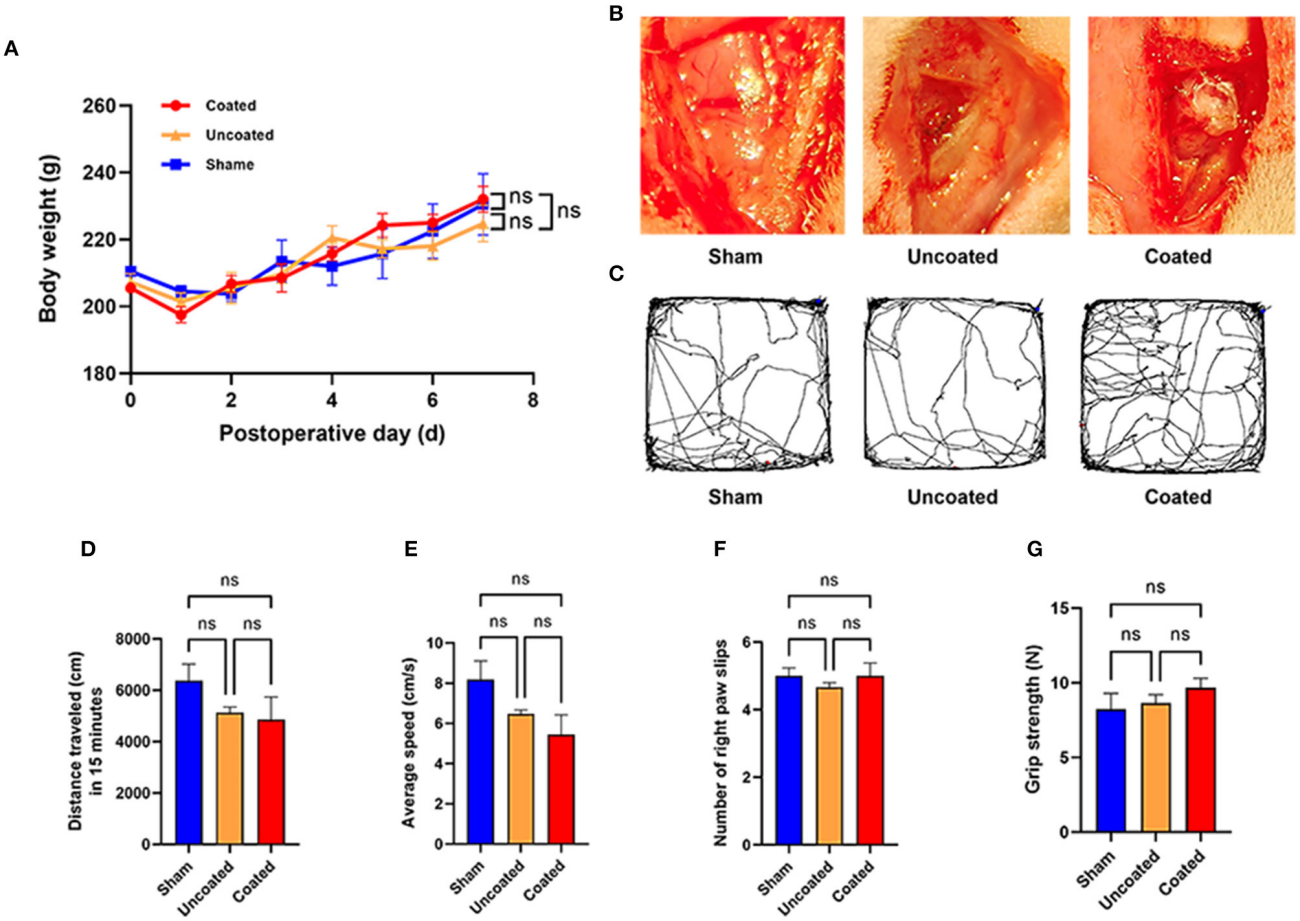
Motor function evaluation of electrocoagulation with BW coated bipolar forceps in rat M1 area. **(A)** Body weight change during 7 days postoperatively. **(B)** Operative view of each experimental group. **(C)** Tracked trajectory of a representative rat in each group during 15 min in the open field test. Total distance traveled **(D)** and average speed **(E)** in the open field test. **(F)** Number of the right paw slips in the ladder test. **(G)** Grip strength of front paws in the grip strength test, *n* = 4 for each group. ANOVA, ns *p* > 0.05.

After electrocoagulation, body weight change did not show a significant difference among sham, electrocoagulation with BW coated or uncoated group (*p* = 0.9605) (Figure 6A). The electrocoagulation conditions were shown in Figure 6B.

In the open field grid test, representative images of the traced line from a rat in each group are shown in Figure 6C. Electrocoagulation with BW coated or uncoated exhibited a similar amount of total traveled distance (ns *p* = 0.2534) (Figure 6D) and average speed (ns *p* = 0.0980) (Figure 6E) as compared with the sham group. Besides, we found that number of right paw slips (ns *p* = 0.6224) (Figure 6F) and grip strength of front paws (ns *p* = 0.4345) (Figure 6G) remained intact in two different electrocoagulation rats. Taken together, we demonstrate that electrocoagulation with BW coated bipolar forceps did not induce abnormalities in motor functions at the 7th day postoperatively.

**Figure 7:**
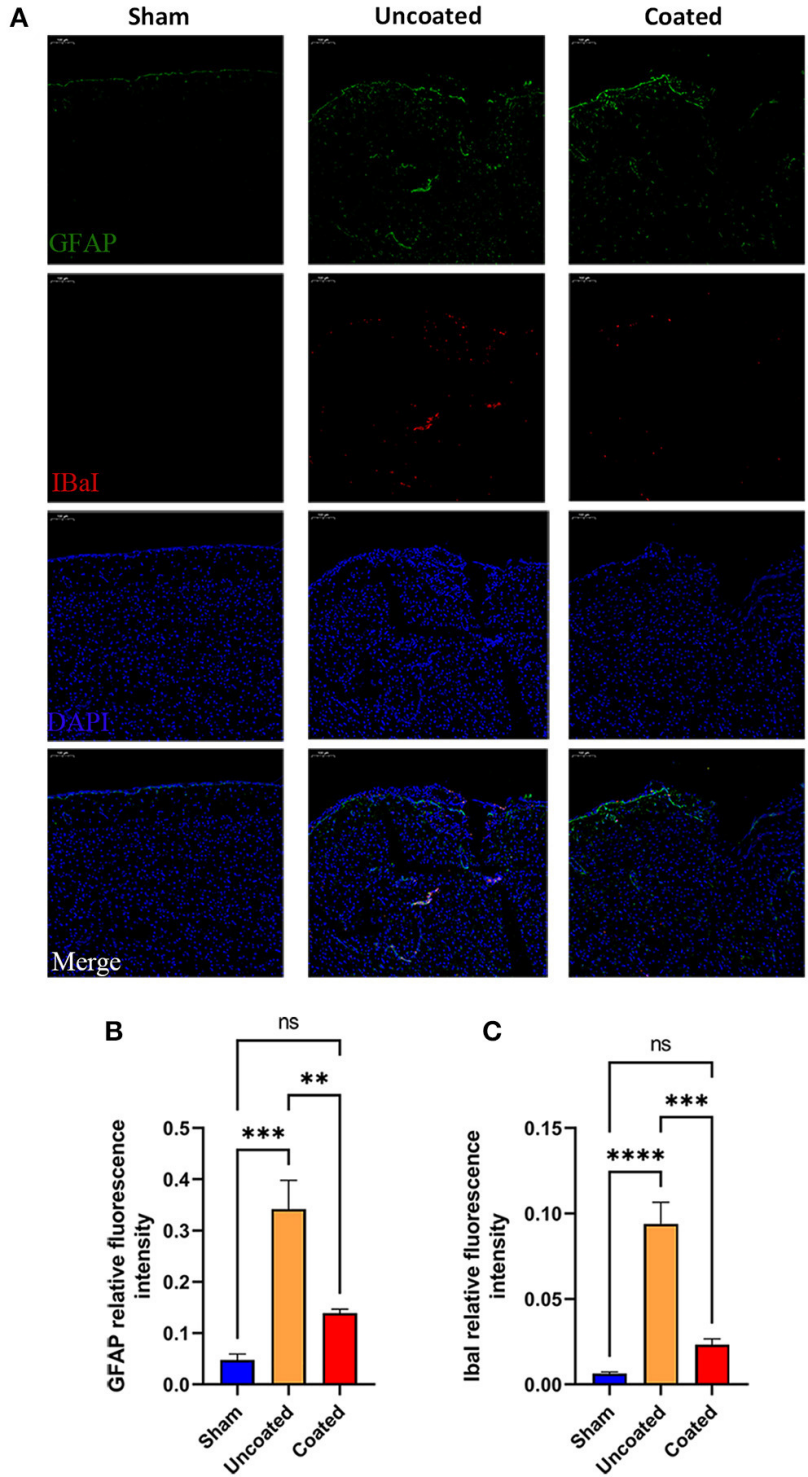
Histology study to determine the inflammation response in different type of electrocoagulation at the 7th day postoperatively. **(A)** Representative fluorescent images (magnification: ×10). IbaI+ **(B)** and GFAP+ **(C)** relative fluorescence intensity in three treatment groups (*n* = 4). ANOVA, ns *p* > 0.05, **p < 0.01, ***p < 0.001, and ****p < 0.0001.

Furthermore, anti-GFAP and anti-IbaI immunofluorescences of each group were performed (Figure 7A). GFAP (Figure 7B) and IbaI (Figure 7C) relative fluorescence intensity in BW coated forceps group has no significant difference compared with the sham group, but much less than the uncoated group (GFAP, coated: 0.1380 ± 0.01178 vs. uncoated: 0.3417 ± 0.05636, ^**^ *p* = 0.0053; IbaI, coated: 0.02335 ± 0.003352 vs. uncoated: 0.09397 ± 0.01260, ^***^ *p* = 0.0003), which indicated that BW coated forceps could significantly ameliorate local inflammation caused by electrocoagulation.

## Discussion

Bipolar electrocoagulation, an essential and necessary technology for intraoperative hemorrhage control in neurosurgery (17), has almost become an indispensable part of all neurosurgery procedures, which makes the hemostasis precise and manipulated easily. Nevertheless, during its application, the excess transferred heat and collateral tissue sticking can cause fatal injuries, which remain as disadvantages needed to be considered (5). To solve these problems, several methods were developed during the evolution of bipolar forceps, such as irrigation and technological advances in forceps design (5, 17). Irrigation was found to be thermo-protective to the neurons during bipolar cautery, which was helpful in the reduction of thermal injury and tissue sticking. Whereas, it makes the electrocoagulation less efficient for affecting the current transduction. Regarding bipolar tip technology, many experiments have been conducted about the tips to overcome the problem of tissue adherence and charring (5, 13). The main concerns about the new bipolar tip technology are cost-effectiveness, cell cytotoxicity, and etc. Another critical concern about bipolar coagulation is hemostatic efficiency. In our neurosurgery department, we found bipolar electrocoagulation was not easy to control bleeding in high blood flow, vascular structure deficiency situations, such as malignant glioma and arteriovenous malformation. Moreover, hemostasis of delicate tissues, such as brainstem and spinal cord, is complicated since it is difficult to keep the balance of function preservation and complete hemostasis. Due to inadequate hemostasis, postoperative blood pressure fluctuations can lead to rebleeding at the surgical site, which can cause devastating consequences.

In this experimental study, we developed a novel bipolar coagulation method, bipolar forceps coated with a small amount of BW, which led to better intraoperative coagulation while minimizing the tissue charring, stickiness, thermal damage, and improving the hemostatic efficiency, thus potentially improving outcomes for neurosurgical procedures. Because of the neither absorbed nor metabolized property of BW, we evaluated the safety of electrocoagulation with BW coated bipolar forceps in rat M1 area of the brain. Excitingly, BW coated forceps did not cause any motor deficiency. Furthermore, the inflammation of the coagulated brain area with BW coated bipolar forceps was similar to the level of the sham group, but significantly lower than the uncoated forceps. Thus, this indicated that BW coated forceps can ameliorate local inflammation caused by electrocoagulation. Possibly owing to the alleviating thermal injuries and tissues sticking, astrocytic and microglia reactivity was significantly suppressed. Therefore, electrocoagulation with a small amount of BW coated bipolar forceps is a better performed hemostatic method, which can be applied to control the complex bleeding more effectively and safely. Moreover, to facilitate the usage of small amount of BW in electrocoagulation, we developed the BW application device in this study. The assembly of this device in clinical is convenient. Therefore, neurosurgeons can apply electrocoagulation with BW coated bipolar forceps easily.

**Figure 8:**
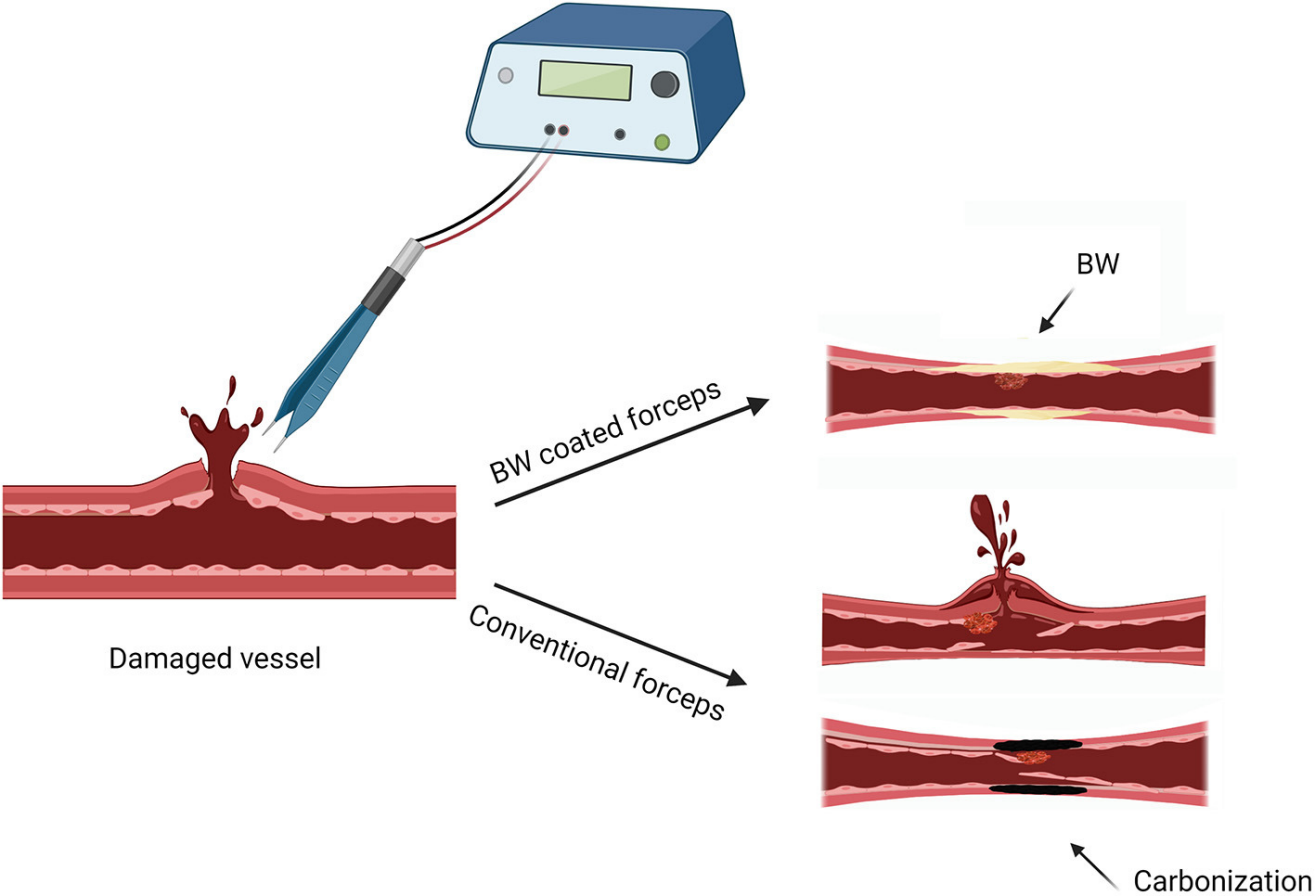
Schematic illustrated the hemostatic mechanism (thermal and mechanical) of electrocoagulation with BW coated bipolar forceps. Created with BioRender.com.

This hemostatic method came about by accident. We found that BW melted by thermal effect of bipolar electrocoagulation can achieve more efficient hemostasis and reduced the amount of BW for stopping bleeding from emissary and diploic veins. The mechanical and thermal dual hemostatic mechanism improved hemostatic efficiency by BW coated bipolar forceps (Figure 8), which verified our finding that melted BW by bipolar electrocoagulation can achieve better hemostasis to bleeding from emissary and diploic veins in clinical applications. Furthermore, because BW is a hydrophobic material that can be melted and peeled off by the thermal effect of electrocoagulation, BW coating, acting as a buffer layer, can reduce adhesion, tissue carbonization, stickiness, and thermal damage. In this study, we kept the damaged CCA clipped until complete hemostasis to assess the hemostatic efficiency of electrocoagulation with BW coated bipolar forceps. Since the conditions requiring hemostasis were the same when electrocoagulation with BW coated and uncoated bipolar forceps, the results can reflect to some extent the hemostatic efficiency of both electrocoagulation methods. Nevertheless, in actual clinical practice, neurosurgeons must perform hemostasis while bleeding is constantly flowing from the damaged tissue. This can be a limitation of the present study.

Currently, several alternatives of BW have been developed (18), none of them are yet a complete replacement for traditional BW for its availability, ease of use, immediate action, and minimal adverse effects. The applications of BW in neurosurgery cover an extensive range, such as hemostasis of bone surfaces, to control bleeding from emissary, and diploic veins in neurosurgery. (19). To facilitate clinical translation, we chose the traditional BW for the whole research. However, it is well known that complications usually accompany the use of traditional BW, such as inflammation, infection, and allergy (18). Thus, usage of BW in a large quantity is not recommended. To the best of our knowledge, little information is available on the brain parenchyma response to BW. Some observations about forming foreign body granuloma comes from several clinical case reports about “BW migration” with the use of BW in a large quantity (10, 11). For electrocoagulation in this study, only a small amount BW was used. We further evaluate the safety of electrocoagulation in M1 area of rats, which demonstrated that application of small amount of BW in rat brain parenchyma was safe for 7 days postoperatively. Since BW is a foreign body, the risk of infection, seizure, and peripheral embolization is worth being aware of. Although the experimental rats did not show suspicious symptoms, the sample size in this study is relatively small to draw a very definite conclusion. More research is needed to evaluate the electrocoagulation with BW in the long term and verified our finding in human beings. Furthermore, with more advanced BW alternatives being developed and tested in clinical, traditional BW can be replaced by the advanced one to improve our current research.

## Conclusion

In this study, we developed a novel bipolar coagulation method: bipolar forceps coated with a small amount of BW, which provides a safe, convenient, cost-efficient, and better performed way for hemostasis. In addition, more research is needed to evaluate electrocoagulation with BW in the long term and verified our finding in human beings.

## Data Availability Statement

The original contributions presented in the study are included in the article/supplementary material, further inquiries can be directed to the corresponding authors.

## Ethics Statement

The animal study was reviewed and approved by the Institutional Animal Care and Use Committee for Zhongnan Hospital of Wuhan University. (Approval No. ZN2021116).

## Author Contributions

JS conceptualized and designed the study, completed the whole experiment, and drafted the initial manuscript. WW designed the study, analyzed the experimental data, and revised the manuscript. ZW helped to finish the animal experiments. HR helped to finish Assessment of Adherence and Thermography and Thermal Damage Assessment related experiments. CJ was in charge of all the photography. LD helped to analyze the experimental data. ZL, JZ, YF, and KH participated in some parts of these experiments. XL conceptualized and designed the study and reviewed and revised the manuscript. JC conceptualized and designed the study, design the bone wax application device, and reviewed and revised the manuscript. All authors approved the final manuscript as submitted and agree to be accountable for all aspects of the work.

## Funding

All phases of this study were supported by the National Natural Science Foundation of China [No. 82001421 (XL)], the Hubei Technological Innovation Special Fund [No. 2018ACA139], and Medical Sci-Tech Innovation Platform of Zhongnan Hospital, Wuhan University [No. PTXM2021006 to XL]. No other Financial Disclosure was involved in this study.

## Conflict of Interest

The authors declare that the research was conducted in the absence of any commercial or financial relationships that could be construed as a potential conflict of interest.

## Publisher's Note

All claims expressed in this article are solely those of the authors and do not necessarily represent those of their affiliated organizations, or those of the publisher, the editors and the reviewers. Any product that may be evaluated in this article, or claim that may be made by its manufacturer, is not guaranteed or endorsed by the publisher.
